# Supplementary material for: Unscheduled DNA replication in G1 causes genome instability and damage signatures indicative of replication collisions
Source: Nat Commun. 2022 Nov 18;13:7014. doi: 10.1038/s41467-022-34379-2 (PMC9674678; doi:10.1038/s41467-022-34379-2)
Supplement: Supplementary file 1 — Supplementary Information [file 41467_2022_34379_MOESM1_ESM.pdf]

# Supplementary Information

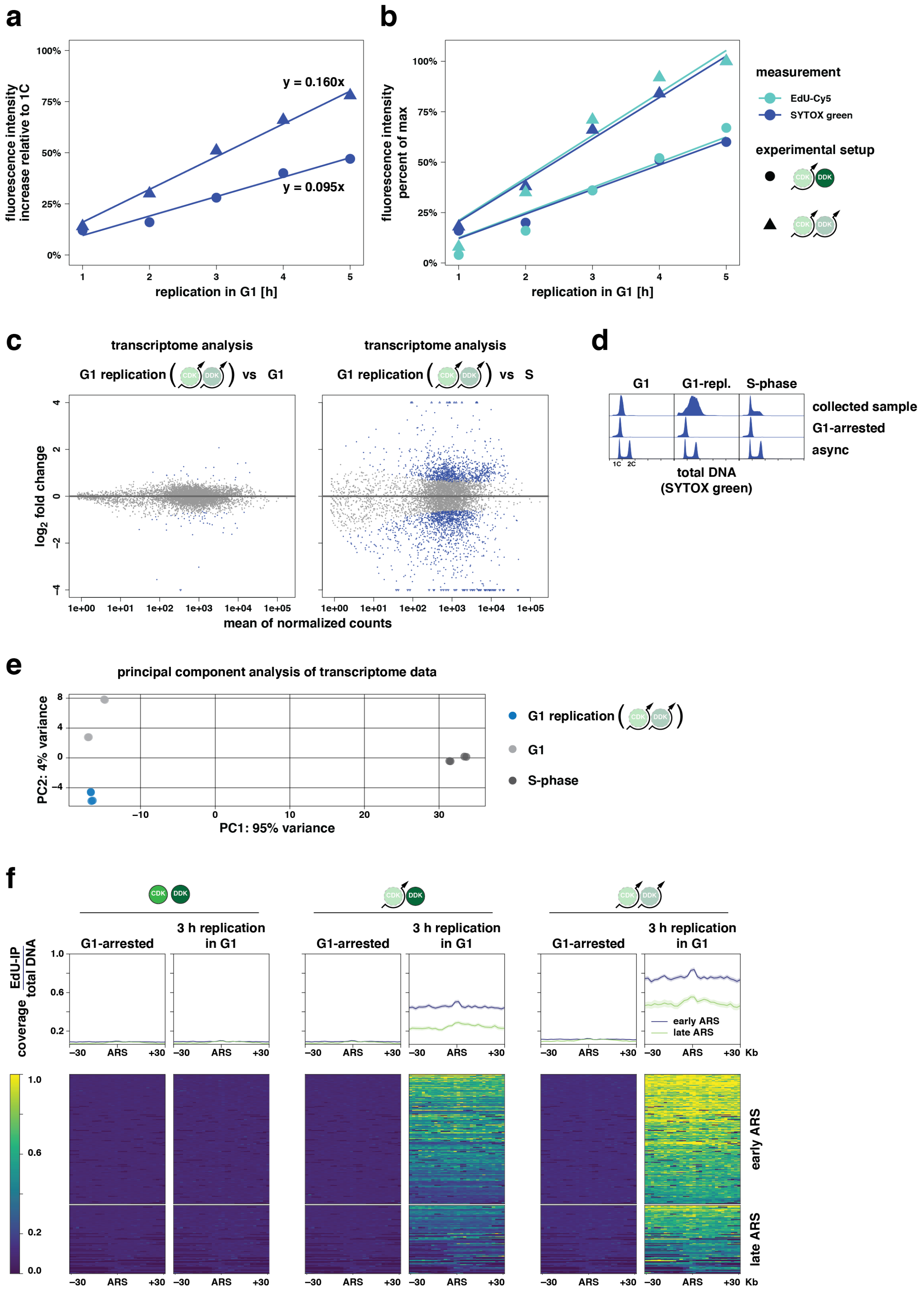

***Supplementary Figure 1***
***Unscheduled replication in G1 initiates at canonical replication origins genome-wide***

(a) DNA content increases linearly during unscheduled replication in G1. Data from Fig. 1b. Cells were arrested in G1 and replication was induced by using either CDK bypass or CDK/DDK bypass systems. Mean fluorescence intensity of total DNA (SYTOX green, blue) was measured by flow cytometry after induction of G1 replication for the indicated amount of time. Data were corrected for background mitochondrial DNA synthesis in the control strain, normalized to a DNA content of 1 C and fitted with a linear regression model (see equation).

(b) DNA is quantitatively labeled with EdU during G1 replication. Data from Fig. 1b. As in (a), additionally measuring mean fluorescence intensity of newly synthesized DNA in G1 (EdU-Cy5, turquoise). Data were corrected for background mitochondrial DNA synthesis in the control strain and normalized to the respective maximum value.

(c) and (d) and (e) Cells keep a G1-like transcriptional signature despite induction of replication and do not transit into an S-like state. Analysis of transcript abundance by mRNA-seq of control cells in G1 and S-phase as well as CDK/DDK bypass cells undergoing unscheduled replication in G1. Control cells were harvested after G1 arrest or released to S-phase; G1-replicating cells were harvested after G1 arrest followed by 3 h of replication induced by CDK/DDK bypass. Poly(A)-containing RNAs were enriched from total RNA and sequenced. (c) Comparison of the transcriptome of CDK/DDK bypass cells in G1 to control cells in G1-phase (left) or S-phase (right). The abundance of individual transcripts is plotted against their fold change in the compared conditions. Transcripts with a statistically significant log-fold change > 0.5 are colored blue. Triangles indicate transcript data that fell outside of the plotted interval. (d) Representative plots of total cellular DNA content as a proxy for cell cycle stage and DNA replication activity. DNA was stained with SYTOX green and measured by flow cytometry. (e) Principal component analysis of the dataset. Cell cycle phase explains 95% of the observed variance between the three conditions. Data from n=4 biological replicates per experimental condition

(f) Early-replicating origins are activated during G1 replication. Related to Fig. 1c. Cells were arrested in G1 and replication was induced for 3 h in the presence of 100 $\mu$M EdU. Input-normalized EdU-sequencing data were analyzed at early or late replication origins (ARS) ± 30 Kb and showed wide-spread replication. (top) Profile plots of mean coverage (dark) ± SE (light). (bottom) Heatmaps with 1 Kb bin size. Data are representative of n=2 biological replicates.

**a**

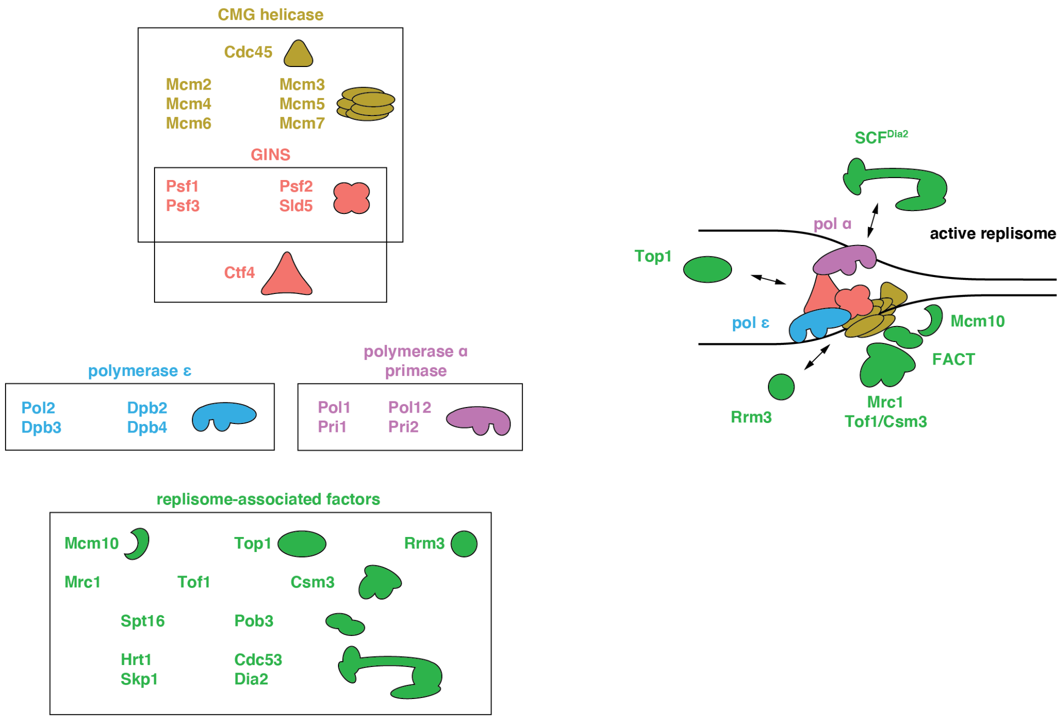


***Supplementary Figure 2***

***Sub-complexes within the replisome***

(a) Schematic drawing of a replisome and its sub-complexes. Color code as in Fig. 2. Interaction of GINS/Ctf4 with Mcm2-7/Cdc45 indicates formation of the CMG helicase, which is the key step in the transition from inactive helicase precursors to active replisomes during replication initiation. DNA polymerases and replisome-associated factors are recruited during or after this transition and travel with the replisome.

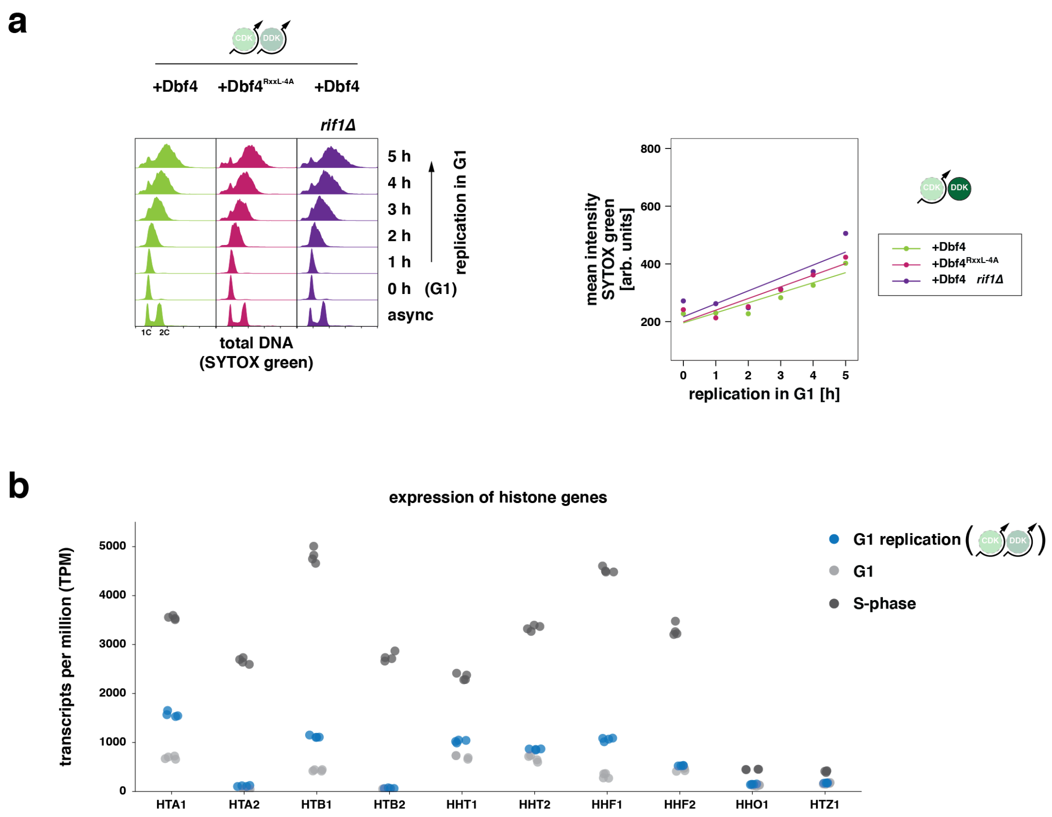


## *Supplementary Figure 3*

## *Factors limiting unscheduled G1 replication*

(a) Enhanced deregulation of Dbf4-dependent kinase (DDK) does not increase the amount of unscheduled replication in G1 under CDK/DDK bypass conditions. Strains expressing high levels of either *DBF4, expressing high levels of* a *DBF4* allele with mutated destruction-boxes (*dbf4*$^{RxxL\text{-}4A}$), or lacking *RIF1* (a targeting subunit for the DDK-antagonizing PP1 phosphatase) were additionally used in this experiment. (left) SYTOX green-stained total DNA as measured by flow cytometry at the indicated timepoints shows highly similar progression of unscheduled DNA replication during G1. (right) Quantification of the flow cytometry data by approximation of a bimodal distribution and calculating the means of the individual normal distributions. The average mean from 5 fits per timepoint is shown together with a linear regression. Data are representative of n=2 biological replicates.

(b) Expression of histone genes is low during G1 replication but strongly induced during early S phase. The frequency of individual histone gene transcripts (transcriptome data as analyzed in Supplementary Figure 1c-e) from cells in G1-phase, S-phase, or undergoing G1 replication, normalized for library size and transcript length. Data from n=4 biological replicates per condition.

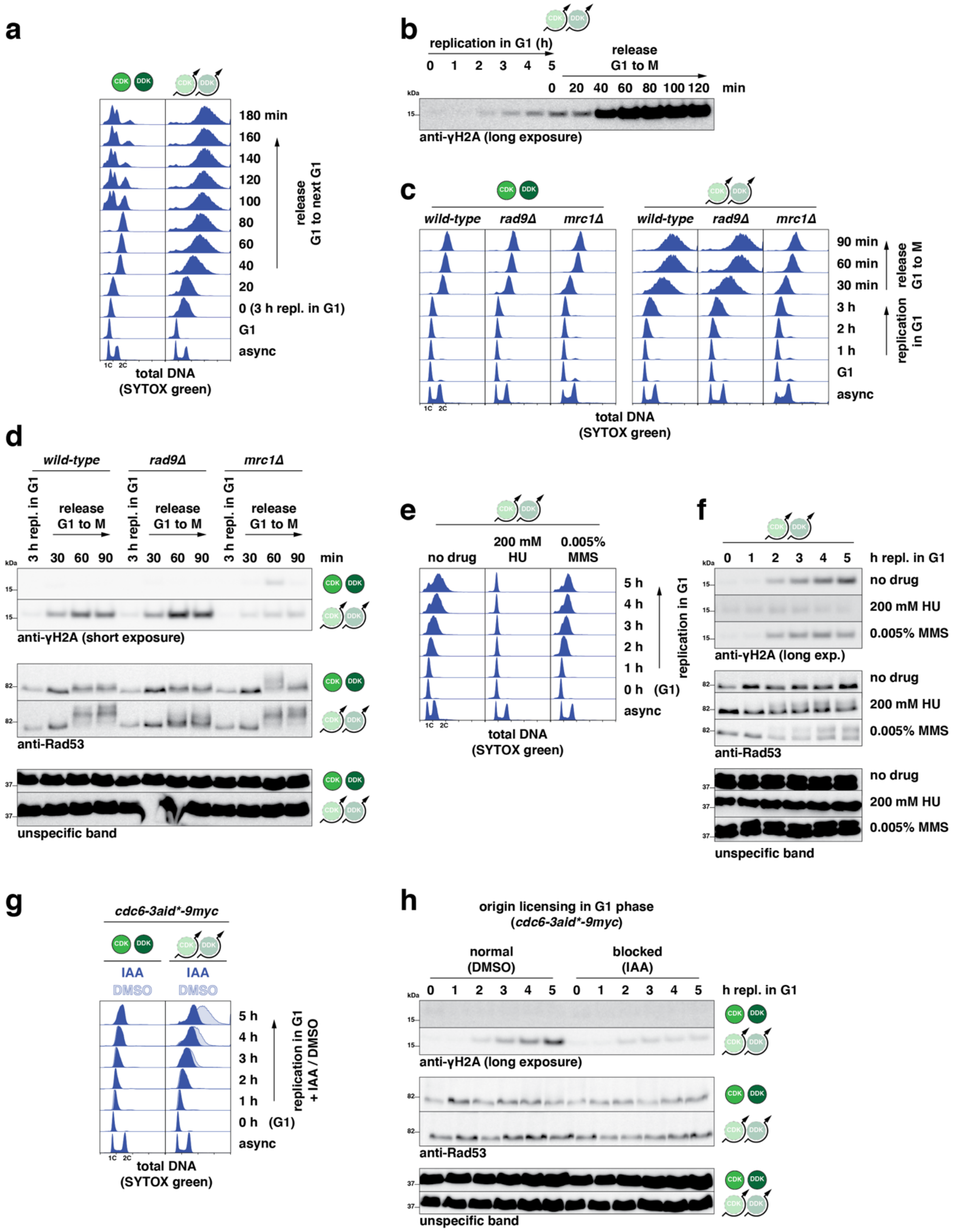
a
CDK DDK
180 min
160
140
120
100
80
60
40
20
0 (3 h repl. in G1)
G1
async
release G1 to next G1
1C 2C
total DNA (SYTOX green)
b
replication in G1 (h)
0 1 2 3 4 5
release G1 to M
0 20 40 60 80 100 120 min
anti-γH2A (long exposure)
c
wild-type rad9Δ mrc1Δ
90 min
60 min
30 min
3 h
2 h
1 h
G1
async
release G1 to M
replication in G1
total DNA (SYTOX green)
d
wild-type rad9Δ mrc1Δ
3 h repl. in G1
release G1 to M
30 60 90 min
anti-γH2A (short exposure)
anti-Rad53
unspecific band
e
no drug
200 mM HU
0.005% MMS
5 h
4 h
3 h
2 h
1 h
0 h (G1)
async
replication in G1
total DNA (SYTOX green)
f
0 1 2 3 4 5 h repl. in G1
no drug
200 mM HU
0.005% MMS
anti-γH2A (long exp.)
anti-Rad53
unspecific band
g
cdc6-3aid*-9myc
IAA
DMSO
5 h
4 h
3 h
2 h
1 h
0 h (G1)
async
replication in G1 + IAA / DMSO
total DNA (SYTOX green)
h
origin licensing in G1 phase (cdc6-3aid*-9myc)
normal (DMSO)
blocked (IAA)
0 1 2 3 4 5 0 1 2 3 4 5 h repl. in G1
anti-γH2A (long exposure)
anti-Rad53
unspecific band

***Supplementary Figure 4***
***Unscheduled G1 replication induces DNA damage upon subsequent S-phase replication***

(a) Release from unscheduled G1 replication results in cell cycle arrest after S-phase. After 3 h of replication in G1 induced by using the CDK/DDK bypass system, cells were released to the cell cycle and arrested in the next G1-phase. Total DNA content (SYTOX green stain) was measured by flow cytometry at the indicated timepoints. Starting at 80 min control cells enter the next G1-phase, while G1 replication cells stay arrested with G2/M DNA content. Data are representative of n=2 biological replicates.

(b) Unscheduled G1 replication generates low amounts of DNA damage already in G1. Longer exposure of the γH2A western blot that is shown in Fig. 4b (representative of n=2 biological replicates).

(c) and (d) DNA damage after unscheduled replication in G1 is detected by the Rad9-dependent DNA damage checkpoint. Unscheduled replication in G1 was induced in cells deficient in Rad9 or Mrc1 checkpoint signaling factors by CDK/DDK bypass before release of the cells to nocodazole-containing medium. (c) SYTOX green-stained total DNA content as measured by flow cytometry at the indicated timepoints. (d) Western blots of samples from (c) detecting γH2A and Rad53 at the indicated timepoints. An unspecific band from the Rad53 western blots is included as a loading control. Data are representative of n=2 biological replicates.

(e) and (f) The checkpoint detects canonical replication stress (replication fork stalling) in G1-arrested cells. G1 replication was induced by the CDK/DDK bypass system, but in the presence of additional replication perturbation by hydroxyurea (HU) or methyl methanesulfonate (MMS). (e) Total DNA content (SYTOX green-stained) was measured by flow cytometry. (f) Western blots of samples from (e) detecting γH2A and Rad53 at the indicated timepoints. An unspecific band from the Rad53 western blots is included as a loading control. Data are representative of n=2 biological replicates.

(g) and (h) Generation of DNA damage in G1 requires continuous origin licensing. Replication was induced in G1-arrested cells by CDK/DDK bypass with/without concomitant depletion of licensing factor Cdc6 via an auxin-inducible degron. (g) Total DNA content (SYTOX green-stained) as measured by flow cytometry at the indicated timepoints. (h) Western blots of samples from (g) detecting γH2A and Rad53 at the indicated timepoints. An unspecific band from the Rad53 western blots is included as a loading control. Data are representative of n=2 biological replicates.

Source data are provided as a Source Data file.

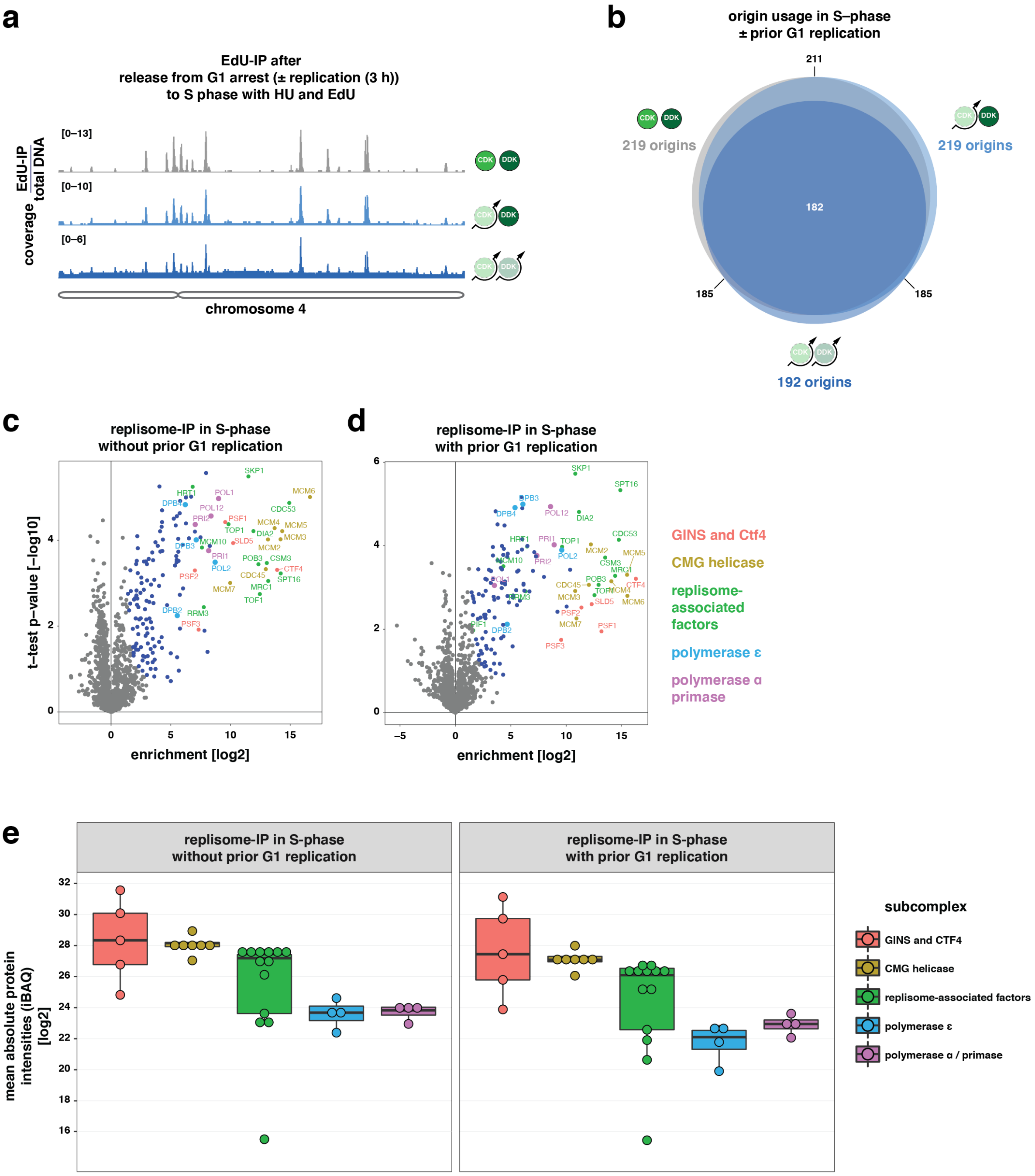
a
EdU-IP after
release from G1 arrest (± replication (3 h))
to S phase with HU and EdU
coverage
EdU-IP
total DNA
[0–13]
[0–10]
[0–6]
CDK
DDK
chromosome 4
b
origin usage in S–phase
± prior G1 replication
211
219 origins
219 origins
182
185
185
192 origins
c
replisome-IP in S-phase
without prior G1 replication
t–test p–value [–log10]
enrichment [log2]
d
replisome-IP in S-phase
with prior G1 replication
enrichment [log2]
GINS and Ctf4
CMG helicase
replisome-associated factors
polymerase ε
polymerase α primase
e
replisome-IP in S-phase
without prior G1 replication
replisome-IP in S-phase
with prior G1 replication
mean absolute protein intensities (iBAQ) [log2]
subcomplex
GINS and CTF4
CMG helicase
replisome-associated factors
polymerase ε
polymerase α / primase

***Supplementary Figure 5***

***G1 replication does not disturb subsequent replication initiation in S-phase***

(a) Unscheduled G1 replication does not affect origin usage in the subsequent S-phase. To visualize origin usage, replication was induced in G1-arrested cells prior to release into S-phase for 90 min in the presence of 200 mM HU and 100 $\mu$M EdU. EdU-labeled DNA was isolated and sequenced. Input-normalized coverage of chromosome 4 in 1 Kb bins is shown as indicated on a linear scale. Y-axes scales are given in the top-left corners. Note the highly similar relative intensity of the peaks and the increased baseline signal in cells after G1 replication which likely is due to ongoing replication from G1 replisomes. Data are representative of n=2 biological replicates.

(b) Unscheduled G1 replication does not affect origin usage in the subsequent S-phase. Data in (a) was used to call peaks and compare between the three experimental conditions. Origins detected in S-phase in control cells are depicted in gray; origins detected in S-phase after CDK and CDK/DDK bypass are depicted in shades of blue and overlap strongly with canonical early origins of S-phase.

(c) to (e) Replisome composition in S-phase is not broadly affected by prior G1 replication. Cells were released from a G1-arrest to S-phase with or without prior induction of replication in G1 by CDK/DDK bypass. Replisomes were affinity-purified from these S-phase extracts via GFP-tagged GINS-subunit Psf2 and analyzed by mass spectrometry. (c)/(d) Volcano-plots of the enrichment of proteins in GFP-tagged samples versus untagged control samples. Colors indicate statistically significantly enriched proteins; different replisome subcomplexes are additionally highlighted as indicated. (e) Label-free quantification and comparison of the datasets shown in (c)/(d) using intensity-based absolute quantification (iBAQ). Boxes indicate the median with the first and third quartile of the sub-complexes, whiskers indicate the minimum and maximum (calculated by extending the box by 1.5 inter-quartile range). Mean iBAQ values for individual proteins are plotted as circles. Data from n=3 biological replicates.

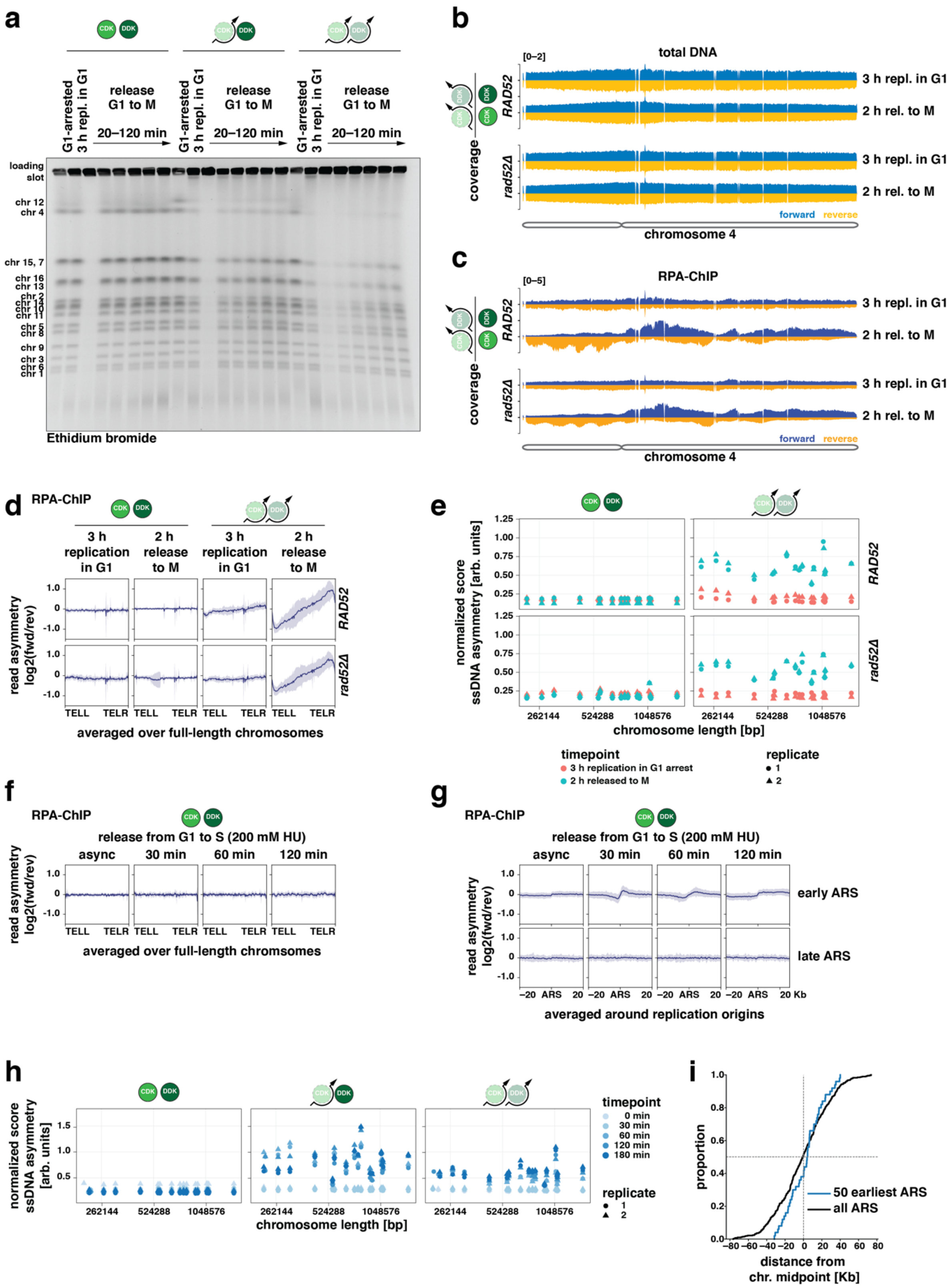
a
G1-arrested
3 h repl. in G1
release
G1 to M
20–120 min
loading
slot
chr 12
chr 4
chr 15, 7
chr 16
chr 13
chr 2
chr 14
chr 10
chr 11
chr 5
chr 8
chr 9
chr 3
chr 6
chr 1
Ethidium bromide
b
total DNA
[0–2]
RAD52
rad52Δ
coverage
3 h repl. in G1
2 h rel. to M
forward
reverse
chromosome 4
c
RPA-ChIP
[0–5]
d
RPA-ChIP
3 h replication in G1
2 h release to M
read asymmetry
log2(fwd/rev)
TELL
TELR
averaged over full-length chromosomes
e
normalized score
ssDNA asymmetry [arb. units]
chromosome length [bp]
timepoint
3 h replication in G1 arrest
2 h released to M
replicate
f
release from G1 to S (200 mM HU)
async
30 min
60 min
120 min
averaged over full-length chromsomes
g
early ARS
late ARS
averaged around replication origins
h
0 min
180 min
i
proportion
50 earliest ARS
all ARS
distance from
chr. midpoint [Kb]

***Supplementary Figure 6***
***Successive G1 and S replication generate single-ended DSBs from head-to-tail-fork collisions, resulting in an asymmetric pattern of RPA-bound ssDNA on chromosome arms***

(a) Persistent replication/repair structures after release from unscheduled G1 replication. Ethidium bromide-stained gel after pulsed-field gel electrophoresis corresponding to Fig. 5a. Samples were taken at the indicated timepoints after inducing G1 replication and released to nocodazole-containing medium and separated on a pulsed-field electrophoresis gel. Data are representative of n=2 biological replicates.

(b) to (e) The strand-biased accumulation of RPA (indicating single-stranded DNA) does not depend on *RAD52* excluding break-induced replication as a causative factor. Cells were arrested in G1, replication was induced for 3 h, and cells were subsequently released to nocodazole-containing medium. Samples were crosslinked at the indicated times and RPA-bound DNA was purified for sequencing. Single-stranded DNA (as indicated by RPA binding) accumulated similarly in the absence and presence of recombination factor Rad52. (b) and (c) show representative coverage traces of reads mapping to chromosome 4 for total DNA (b) and RPA-ChIP (c) of strains with and without *RAD52*. The scale of the y-axes is indicated in the top-left corner of the panels. (d) The read asymmetry (log2-ratio of RPA-ChIP-seq reads mapping to forward and reverse strands) was averaged over full-length chromosomes at the indicated timepoints and plotted as mean (dark) ± SD (light). (e) single-stranded DNA (ssDNA) asymmetry scores for individual chromosomes were calculated by normalizing the log2-ratios of RPA-ChIP-reads mapping to forward and reverse strand in 50 bp bins for chromosome length. Data are representative of n=2 biological replicates.

(e) and (g) Stalled replication forks cause a differential signature of single-stranded DNA accumulating with strand bias around early-replicating origins. RPA-ChIP samples were taken from G1-arrested *wild-type* cells released to S-phase in the presence of 200 mM HU for the indicated times. (f) Read asymmetry (as in (d), plotted as mean (dark) ± SD (light)) was averaged over full-length chromosomes and shows no strand bias. (g) Read asymmetry (as in (d)/(f), plotted as mean (dark) ± SD (light)) but averaged around early- and late-replicating origins (autonomously replicating sequences (ARS)) ± 20 Kb shows a minor strand bias close to replication origins consistent with exposure of ssDNA during lagging strand synthesis.

(h) Asymmetric accumulation of RPA (indicating single-stranded DNA) occurs on both long and short chromosomes. Single-stranded DNA (ssDNA) asymmetry scores for individual chromosomes were calculated for the data in Fig. 5b-g by normalizing log2-ratios of RPA-ChIP-reads mapping to forward and reverse strand at the indicated timepoints in 50 bp bins for chromosome length. Data are representative of n=2 biological replicates.

(i) Early firing origins are preferentially located close to the chromosome midpoint and have a slight skew toward the right part of chromosomes. The distances of either all or the 50 earliest-firing origins from the arithmetic midpoint of each chromosome were plotted as an empirical cumulative density function. Dotted lines indicate the chromosome midpoint (vertical) and the 50% fraction (horizontal).

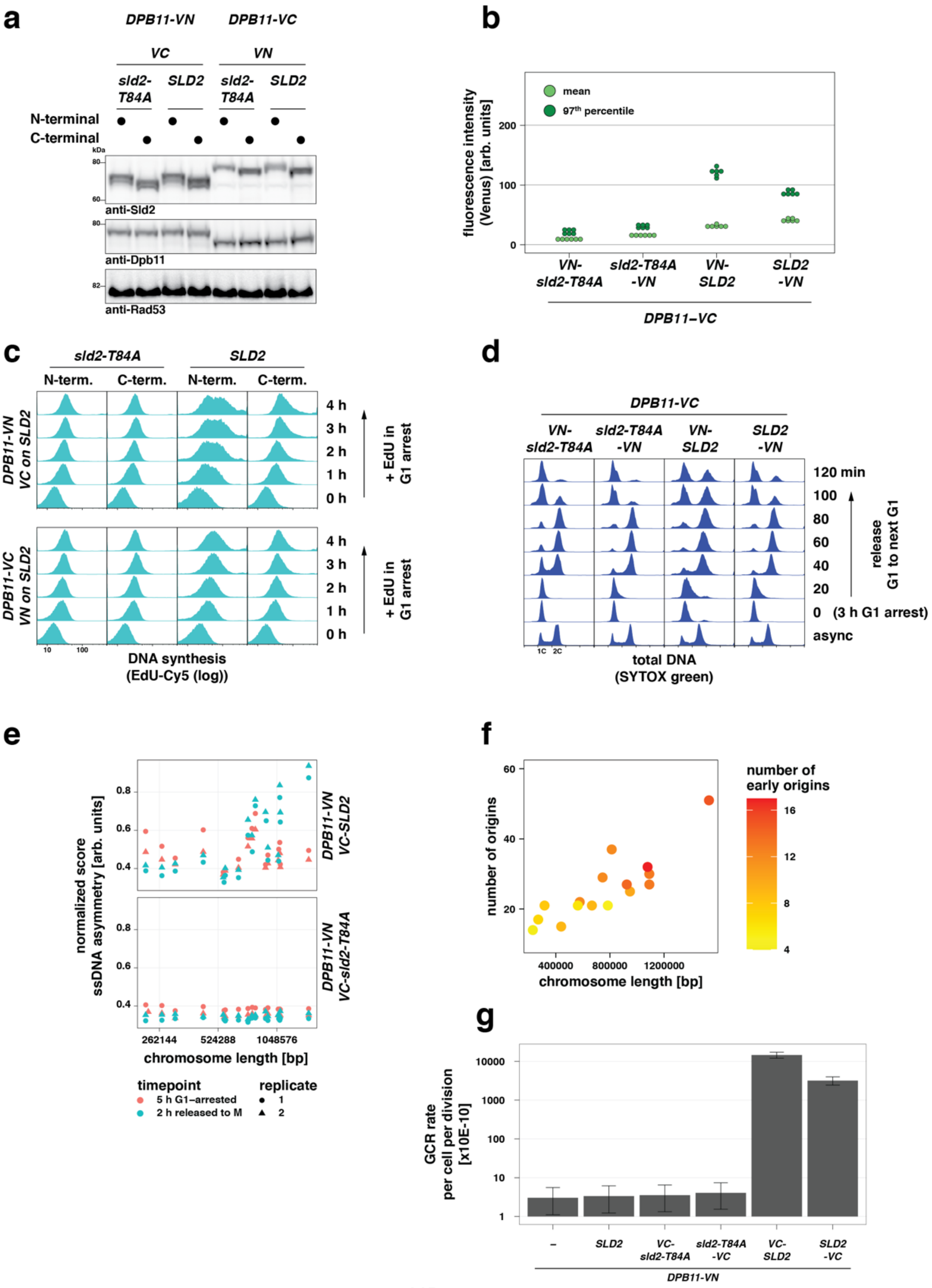


| | | | | | | |
|---|---|---|---|---|---|---|
| GCR rate per cell per division [x10E-10] | 3.0 | 3.3 | 3.5 | 4.1 | 14560.2 | 3176.5 |
| 95% confidence interval for GCR rate | 1.1 – 5.6 | 1.2 – 6.2 | 1.3 – 6.5 | 1.5 – 7.4 | 12042.2 – 17251.1 | 2432.7 – 3991.4 |

***Supplementary Figure 7***

***Low levels of sporadic G1 replication generate head-to-tail fork collisions and genome instability***

(a) Split-Venus tagged Dpb11- and Sld2-constructs are expressed to similar levels. Expression levels of Sld2 and Dpb11 carrying split-Venus tags as well as phosphorylated Rad53 detected by western blots from log-phase samples. Note the faint signal for phosphorylated Rad53 with constructs that stabilize the physical interaction between Dpb11 and Sld2 (*VC-SLD2* and *VN-SLD2*).

(b) Split-Venus tags (VN/VC) stabilize the physical interaction between Dpb11 and Sld2. Dpb11-VC and Sld2 tagged at either N- or C-terminus with VN-fragment of the fluorescent protein Venus. Mean (light green) and 97$^{th}$ percentile (dark green) of split-Venus fluorescence intensity were measured by flow cytometry in log-phase cells. Data from n=6 biological replicates.

(c) Venus-stabilized interaction of Dpb11 and Sld2 induces DNA replication in G1. Cells of the indicated genotypes were pre-arrested in G1 for 1 h and then kept arrested in G1 in the presence of EdU (100 $\mu$M). Incorporated EdU was labeled with Cy5 and measured by flow cytometry. Note the logarithmic scale of the x-axis to resolve different amounts of G1 replication. Data are representative of n=2 biological replicates.

(d) Venus-stabilized interaction of Dpb11 and Sld2 results in cell cycle arrest. Additional samples to experiment shown in Fig. 6b. SYTOX green-stained total DNA from samples at the indicated timepoints after release from G1 arrest to the next G1-phase as measured by flow cytometry.

(e) Sporadic G1 replication affects long chromosomes more strongly. Single-stranded DNA (ssDNA) asymmetry scores for individual chromosomes were calculated by normalizing the log2-ratios of RPA-ChIP-seq reads mapping to forward and reverse strand in 50 bp bins for chromosome length. Data are representative of n=2 biological replicates.

(f) Long chromosomes harbor more early-firing origins. Length of chromosomes was plotted against the total number of ARS sequences. Color intensity indicates the number of early-firing origins.

(g) High levels of genome instability are caused by Venus-stabilized interaction of Dpb11 and Sld2. GCR rates (median) for the assay shown in Fig. 6f were calculated from n=8 cultures by fluctuation analysis. Error bars indicate a 95% confidence interval for the determined GCR rate. Note the logarithmic scaling of the y-axis.

Source data are provided as a Source Data file.

**Fig. 4b (Rad53)**

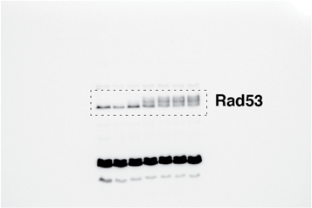


**Fig. 4b (γH2A)**

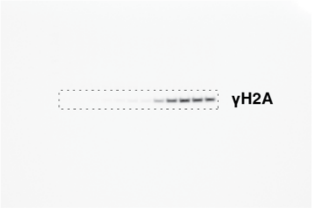


**Supp. Fig. 4b (γH2A)**
**same image as Fig. 4b**
**levels adjusted to show weaker bands**

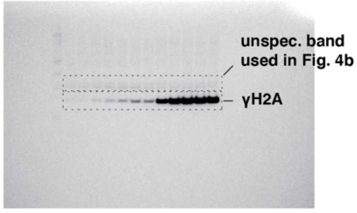


**Fig. 4d (Rad53, control)**

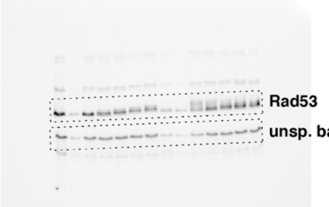


**Fig. 4d (Rad53, CDK/DDK bypass)**

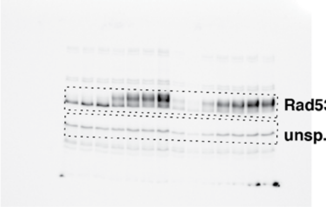


**Fig. 4d (γH2A, control)**

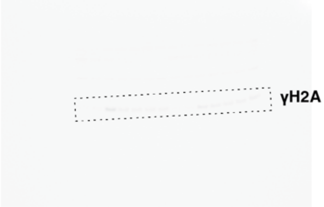


**Fig. 4d (γH2A, CDK/DDK bypass)**

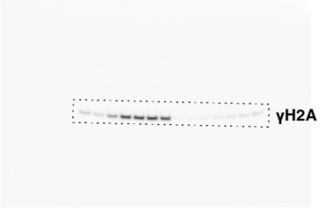


**Fig. 4f (Rad53, control)**

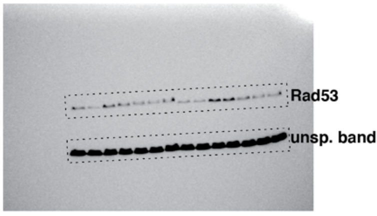


**Fig. 4f (Rad53, CDK/DDK bypass)**

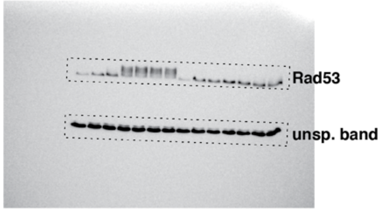


**Fig. 4f (γH2A)**

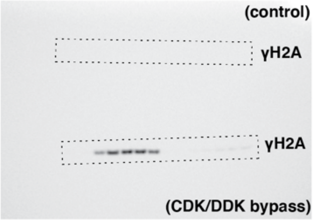


**Supp. Fig. 4d (γH2A, control)**

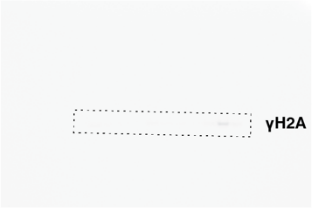


**Supp. Fig. 4d (γH2A, CDK/DDK bypass)**

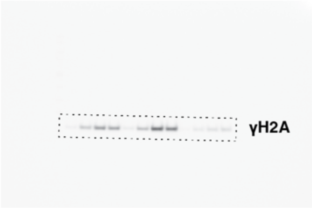


**Supp. Fig. 4d (Rad53, control)**

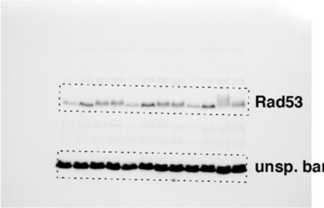


**Supp. Fig. 4d (Rad53, CDK/DDK bypass)**

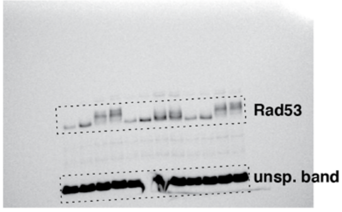

Supp. Fig. 4f (Rad53, no drug)

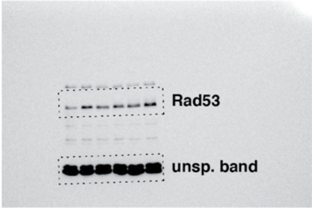


Supp. Fig. 4f (Rad53, 200 mM HU)

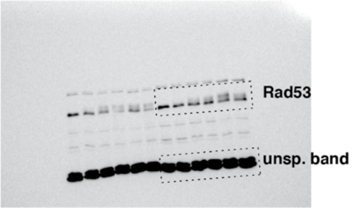


Supp. Fig. 4f (Rad53, 0.005% MMS)

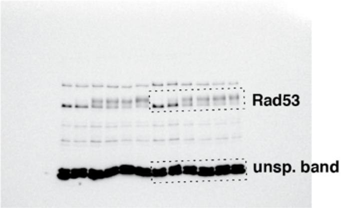


Supp. Fig. 4f (γH2A, no drug)

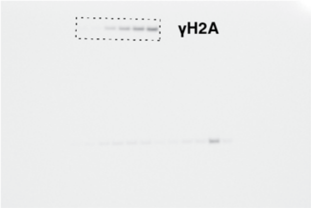


Supp. Fig. 4f (γH2A, 200 mM HU)

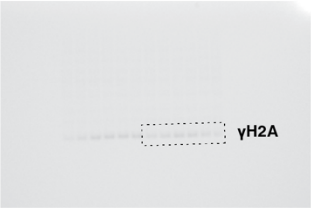


Supp. Fig. 4f (γH2A, 0.005% MMS)

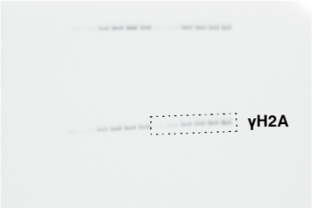


Supp. Fig. 4h (Rad53, control)

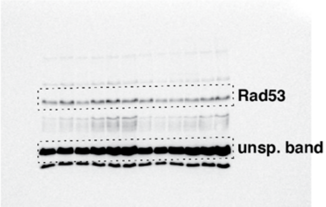


Supp. Fig. 4h (Rad53, CDK/DDK bypass)

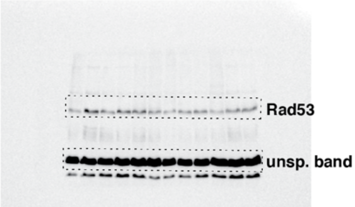


Supp. Fig. 4h (γH2A, control)

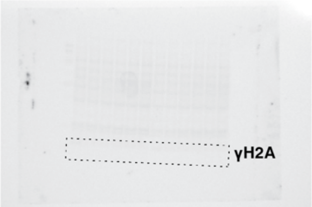


Supp. Fig. 4h (γH2A, CDK/DDK bypass)

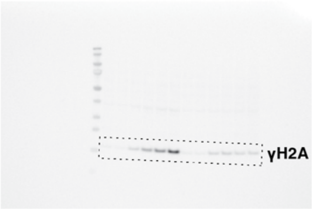


Fig. 6c (Rad53, SLD2-VC)

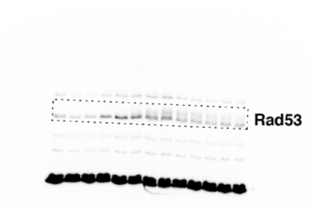


Fig. 6c (Rad53, sld2-T84A-VC)

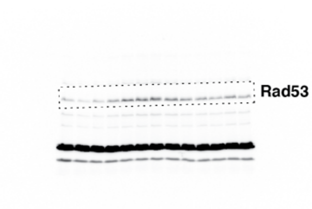


Fig. 6c (Rad53, VC-SLD2)

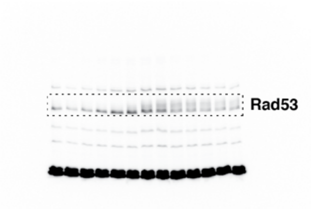


Fig. 6c (Rad53, VC-sld2-T84A)

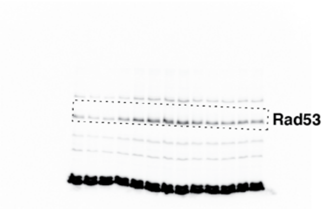


Supp. Fig. 7a (Sld2)

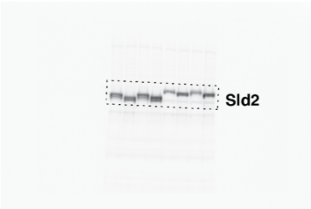


Supp. Fig. 7a (Dpb11)

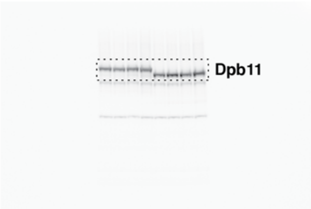


Supp. Fig. 7a (Rad53)

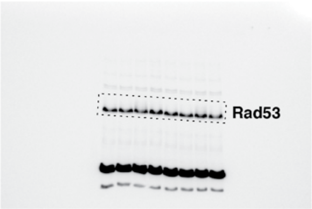


***Supplementary Figure 8***

***Uncropped images of all western blots***

*Boxes indicate the bands shown in the other figures.*

# Supplementary Tables

### *Supplementary Table 1: Budding yeast strains used in this study*

| strain | genotype | source / reference |
|---|---|---|
| W303-1A | *MAT**a** ade2-1 ura3-1 his3-11,15 trp1-1 leu2-3,112 can1-100* | (Rothstein, 1983) |
| E3087 | W303-1A *RAD5+*<br>*ura3::URA3/pGPD-TK(5x)*<br>*AUR1c::pADH-hENT1* | (Talarek et al., 2015) |
| YKR1445 | *leu2::pGAL-DPB11/sld2-T84D::LEU2* | this study |
| YKR1447 | *his3::pGAL-DBF4::HIS3*<br>*leu2::pGAL-DPB11/sld2-T84D::LEU2* | this study |
| YKR1500 | *AUR1c::pADH-hENT1*<br>*bar1Δ::natNT2* | this study |
| YKR1501 | *leu2::pGAL-DPB11/SLD2::LEU2*<br>*bar1Δ::natNT2* | this study |
| YKR1502 | *leu2::pGAL-DPB11/sld2-T84D::LEU2*<br>*bar1Δ::natNT2* | this study |
| YKR1503 | *his3::pGAL-DBF4::HIS3*<br>*leu2::pGAL-DPB11/sld2-T84D::LEU2*<br>*bar1Δ::natNT2* | this study |
| YKR1546 | *pep4Δ::kanMX4* | this study |
| YKR1558 | *pep4Δ::kanMX4*<br>*PSF2-yeGFP::hphNT1* | this study |
| YKR1553 | *pep4Δ::kanxM4*<br>*his3::pGAL-DBF4::HIS3*<br>*leu2::pGAL-DPB11/sld2-T84D::LEU2*<br>*bar1Δ::natNT2* | this study |
| YKR1557 | *pep4Δ::kanxM4*<br>*his3::pGAL-DBF4::HIS3*<br>*leu2::pGAL-DPB11/sld2-T84D::LEU2*<br>*bar1Δ::natNT2 PSF2-yeGFP::hphNT1* | this study |
| YKR1549 | *pep4Δ::kanMX4*<br>*his3::pGAL-DBF4::HIS3*<br>*leu2::pGAL-DPB11/sld2-T84D::LEU2* | this study |
| YKR1615 | *pep4Δ::kanMX4*<br>*his3::pGAL-DBF4::HIS3*<br>*leu2::pGAL-DPB11/sld2-T84D::LEU2*<br>*PSF2-yeGFP::hphNT1* | this study |
| YKR1815 | *leu2::pGAL-DPB11/sld2-T84D::LEU2*<br>*bar1Δ::natNT2*<br>*his3::pGAL-DBF4/SLD3::HIS3*<br>*trp1::pGAL-CDC45/SLD7::TRP1* | this study |
| YKR2113 | *leu2::pGAL-DPB11/sld2-T84D::LEU2*<br>*bar1Δ::natNT2*<br>*his3::pGAL-DBF4::HIS3*<br>*trp1::pGAL-JET1::TRP1* | this study |
| YKR2114 | *leu2::pGAL-DPB11/sld2-T84D::LEU2*<br>*bar1Δ::natNT2*<br>*his3::pGAL-DBF4/SLD3::HIS3*<br>*trp1::pGAL-JET1/SLD7::TRP1* | this study |

| | | |
|---|---|---|
| YKR2035 | *leu2::pGAL-DPB11/sld2-T84D::LEU2*<br>*bar1Δ::natNT2*<br>*his3::pGAL-DBF4/SPT21-3FLAG::HIS3* | this study |
| YKR2090 | *his3::pGAL-DBF4::HIS3*<br>*leu2::pGAL-DPB11/sld2-T84D::LEU2*<br>*bar1Δ::natNT2*<br>*cdc45::JET1::TRP1* | this study |
| YKR2108 | *leu2::pGAL-DPB11/sld2-T84D::LEU2*<br>*bar1Δ::natNT2*<br>*cdc45::JET1::TRP1*<br>*his3::pGAL-DBF4/SPT21-3FLAG::HIS3* | this study |
| YKR1603 | *leu2::pGAL-DPB11/sld2-T84D::LEU2*<br>*bar1Δ::natNT2*<br>*his3::pGAL-dbf4-RxxL-4A (R10A,L13A,R62A,L65A)::HIS3* | this study |
| YKR1563 | *his3::pGAL-DBF4::HIS3*<br>*leu2::pGAL-DPB11/sld2-T84D::LEU2*<br>*bar1Δ::natNT2*<br>*rif1Δ::hphNT1* | this study |
| YKR1803 | *his3::pGAL-DBF4::HIS3*<br>*leu2::pGAL-DPB11/sld2-T84D::LEU2*<br>*bar1Δ::natNT2*<br>*sml1Δ::hphNT1*<br>*dif1Δ::kanMX4* | this study |
| YKR1614 | *leu2::pGAL-DPB11/sld2-T84D::LEU2*<br>*his3::pGAL-DBF4/RNR1::HIS3*<br>*bar1Δ::natNT2* | this study |
| YKR1824 | *leu2::pGAL-DPB11/sld2-T84D::LEU2*<br>*bar1Δ::natNT2*<br>*his3::pGAL-DBF4/rnr1-D57N::HIS3* | this study |
| YKR2099 | *his3::pGAL-DBF4::HIS3*<br>*leu2::pGAL-DPB11/sld2-T84D::LEU2*<br>*bar1Δ::natNT2*<br>*trp1::pGAL-CDC6::TRP1* | this study |
| YKR2059 | *trp1::pTDH3-TIR1-9myc,tTA,tetR'-SSN6::TRP1*<br>*SLD3::(hphNT1)iAID-promoter-sld3-3aid*-9myc::natNT2* | this study |
| YKR2067 | *leu2::pGAL-DPB11/sld2-T84D::LEU2*<br>*his3::pGAL-DBF4::HIS3*<br>*trp1::pTDH3-TIR1-9myc,tTA,tetR'-SSN6::TRP1*<br>*SLD3::(hphNT1)iAID-promoter-sld3-3aid*-9myc::natNT2* | this study |
| YKR1538 | *trp1::DDC1-RAD9-3FLAG::TRP1* | this study |
| YKR1541 | *his3::pGAL-DBF4::HIS3*<br>*leu2::pGAL-DPB11/sld2-T84D::LEU2*<br>*trp1::DDC1-RAD9-3FLAG::TRP1* | this study |
| YKR1564 | *trp1::DDC1-RAD9-3FLAG::TRP1*<br>*bar1Δ::natNT2* | this study |
| YKR1567 | *his3::pGAL-DBF4::HIS3*<br>*leu2::pGAL-DPB11/sld2-T84D::LEU2*<br>*trp1::DDC1-RAD9-3FLAG::TRP1*<br>*bar1Δ::natNT2* | this study |
| YKR2025 | *rad9Δ::hphNT1* | this study |
| YKR2026 | *mrc1Δ::hphNT1* | this study |
| YKR1484 | *his3::pGAL-DBF4::HIS3*<br>*leu2::pGAL-DPB11/sld2-T84D::LEU2*<br>*rad9Δ::hphNT1* | this study |

| | | |
|---|---|---|
| YKR1481 | *his3::pGAL-DBF4::HIS3*<br>*leu2::pGAL-DPB11/sld2-T84D::LEU2*<br>*mrc1Δ::hphNT1* | this study |
| YKR1996 | *trp1::pGPD-TIR1-3myc::TRP1*<br>*cdc6-3aid*-9myc::natNT2* | this study |
| YKR2000 | *trp1::pGPD-TIR1-3myc::TRP1*<br>*leu2::pGAL-DPB11/sld2-T84D::LEU2*<br>*his3::pGAL-DBF4::HIS3*<br>*cdc6-3aid*-9myc::natNT2* | this study |
| YKR1754 | *ARS702::[I-SceI-cut site]::TRP1* | this study |
| YKR1755 | *leu2::pGAL-DPB11/sld2-T84D::LEU2*<br>*ARS702::[I-SceI-cut site]::TRP1* | this study |
| YKR1756 | *his3::pGAL-DBF4::HIS3*<br>*leu2::pGAL-DPB11/sld2-T84D::LEU2*<br>*ARS702::[I-SceI-cut site]::TRP1* | this study |
| YKR1642 | *rad52Δ::hphNT1* | this study |
| YKR1645 | *his3::pGAL-DBF4::HIS3*<br>*leu2::pGAL-DPB11/sld2-T84D::LEU2*<br>*rad52Δ::hphNT1* | this study |
| YKR1737 | *leu2::pADH-DPB11-VN::LEU2* | this study |
| YKR1768 | *leu2::pADH-DPB11-VN::LEU2*<br>*trp1::pADH-VC-sld2-T84A::TRP1* | this study |
| YKR1832 | *leu2::pADH-DPB11-VN::LEU2*<br>*trp1::pADH-sld2-T84A-VC::TRP1* | this study |
| YKR1767 | *leu2::pADH-DPB11-VN::LEU2*<br>*trp1::pADH-VC-SLD2::TRP1* | this study |
| YKR1831 | *leu2::pADH-DPB11-VN::LEU2*<br>*trp1::pADH-SLD2-VC::TRP1* | this study |
| YKR1830 | *leu2::pADH-DPB11-VC::LEU2* | this study |
| YKR1836 | *leu2::pADH-DPB11-VC::LEU2*<br>*trp1::pADH-VN-sld2-T84A::TRP1* | this study |
| YKR1834 | *leu2::pADH-DPB11-VC::LEU2*<br>*trp1::pADH-sld2-T84A-VN::TRP1* | this study |
| YKR1835 | *leu2::pADH-DPB11-VC::LEU2*<br>*trp1::pADH-VN-SLD2::TRP1* | this study |
| YKR1833 | *leu2::pADH-DPB11-VC::LEU2*<br>*trp1::pADH-SLD2-VN::TRP1* | this study |
| YKR1763 | W303-1A *CAN1::URA3*<br>*leu2::pADH-DPB11-VN::LEU2* | this study |
| YKR1783 | W303-1A *CAN1::URA3*<br>*leu2::pADH-DPB11-VN::LEU2*<br>*trp1::pADH-SLD2::TRP1* | this study |
| YKR1780 | W303-1A *CAN1::URA3*<br>*leu2::pADH-DPB11-VN::LEU2*<br>*trp1::pADH-VC-SLD2::TRP1* | this study |
| YKR1781 | W303-1A *CAN1::URA3*<br>*leu2::pADH-DPB11-VN::LEU2*<br>*trp1::pADH-VC-sld2-T84A::TRP1* | this study |
| YKR1850 | W303-1A *CAN1::URA3*<br>*leu2::pADH-DPB11-VN::LEU2*<br>*trp1::pADH-SLD2-VC::TRP1* | this study |

| | | |
|---|---|---|
| YKR1851 | W303-1A *CAN1::URA3 leu2::pADH-DPB11-VN::LEU2 trp1::pADH-sld2-T84A-VC::TRP1* | this study |

***Supplementary Table 2: Plasmids used in this study***

| **plasmid name** | **vector** | **insert** |
|---|---|---|
| pKR588 | pFA6a-natNT2 | *3aid*-9myc* |
| pKR534 | YIplac128 | *pGAL-DPB11/SLD2* |
| pKR535 | YIplac128 | *pGAL-DPB11/sld2-T84D* |
| pKR520 | pRS303 | *pGAL-DBF4* |
| pKR546 | pRS303 | *pGAL-DBF4/SLD3* |
| pKR581 | YIplac204 | *pGAL-CDC45/SLD7* |
| pKR609 | YIplac204 | *pGAL-JET1/SLD7* |
| pKR608 | YIplac204 | *pGAL-JET1* |
| pKR598 | pRS303 | *pGAL-SPT21-3FLAG* |
| pKR592 | pRS303 | *pGAL-DBF4/SPT21-3FLAG* |
| pKR604 | YIplac204 | *pGAL-PRI1/PRI2* |
| pKR614 | pRS303 | *pGAL-POL1-3FLAG/POL12* |
| pKR531 | pRS303 | *pGAL-dbf4ΔD-box* |
| pKR563 | pRS303 | *pGAL-RNR1* |
| pKR582 | pRS303 | *pGAL-rnr1-D57N* |
| pKR562 | pRS303 | *pGAL-DBF4/RNR1* |
| pKR583 | pRS303 | *pGAL-DBF4/rnr1-D57N* |
| pKR508 | YIplac204 | *pGAL-CDC6* |
| pKR548 | pRS304 | *pGPD-TIR1-3myc* |
| pKR385 | YIplac128 | *pADH-DPB11-VN* |
| pKR386 | YIplac128 | *pADH-DPB11-VC* |
| pKR477 | YIplac204 | *pADH-SLD2* |
| pKR417 | YIplac204 | *pADH-VC-SLD2* |
| pKR425 | YIplac204 | *pADH-VC-sld2-T84A* |
| pKR403 | YIplac204 | *pADH-SLD2-VC* |
| pKR413 | YIplac204 | *pADH-sld2-T84A-VC* |
| pKR416 | YIplac204 | *pADH-VN-SLD2* |
| pKR424 | YIplac204 | *pADH-VN-sld2-T84A* |
| pKR402 | YIplac204 | *pADH-SLD2-VN* |
| pKR412 | YIplac204 | *pADH-sld2-T84A-VN* |

## *Supplementary Table 3: Fold changes of individual replisome proteins*

| id | genes | complex | LFQ G1_1 | LFQ G1_2 | LFQ G1_3 | LFQ S_1 | LFQ S_2 | LFQ S_3 | mean LFQ G1 | mean LFQ S | fold change S vs G1 | p-value |
|---|---|---|---|---|---|---|---|---|---|---|---|---|
| P40359 | PSF2 | GINS and CTF4 | 37.857 | 38.068 | 38.269 | 37.943 | 37.760 | 37.852 | 37.852 | 38.065 | -0.213 | 0.17734 |
| Q01454 | CTF4 | GINS and CTF4 | 39.443 | 39.456 | 39.641 | 39.099 | 39.027 | 39.257 | 39.128 | 39.513 | -0.386 | 0.01456 |
| Q03406 | SLD5 | GINS and CTF4 | 38.440 | 38.282 | 38.764 | 38.012 | 38.086 | 38.175 | 38.091 | 38.496 | -0.405 | 0.05386 |
| Q12146 | PSF3 | GINS and CTF4 | 37.505 | 37.600 | 37.110 | 37.074 | 37.066 | 36.485 | 36.875 | 37.405 | -0.530 | 0.09762 |
| Q12488 | PSF1 | GINS and CTF4 | 38.108 | 37.949 | 37.853 | 37.593 | 37.456 | 37.810 | 37.620 | 37.970 | -0.351 | 0.05097 |
| P24279 | MCM3 | CMG helicase | 34.585 | 34.824 | 35.174 | 37.001 | 37.067 | 36.797 | 36.955 | 34.861 | 2.094 | 0.00038 |
| P29469 | MCM2 | CMG helicase | 34.357 | 34.169 | 34.596 | 36.597 | 36.987 | 36.893 | 36.826 | 34.374 | 2.452 | 0.00014 |
| P29496 | MCM5 | CMG helicase | 34.729 | 34.210 | 34.005 | 37.063 | 36.840 | 36.706 | 36.870 | 34.315 | 2.555 | 0.00044 |
| P30665 | MCM4 | CMG helicase | 35.145 | 34.891 | 35.110 | 37.366 | 37.150 | 37.322 | 37.279 | 35.049 | 2.230 | 0.00003 |
| P38132 | MCM7 | CMG helicase | 34.661 | 34.346 | 34.574 | 36.786 | 36.846 | 36.768 | 36.800 | 34.527 | 2.273 | 0.00002 |
| P53091 | MCM6 | CMG helicase | 35.001 | 34.633 | 34.829 | 36.960 | 37.223 | 37.071 | 37.085 | 34.821 | 2.263 | 0.00007 |
| Q08032 | CDC45 | CMG helicase | 32.307 | 33.600 | 32.897 | 35.956 | 35.797 | 35.989 | 35.914 | 32.935 | 2.979 | 0.00140 |
| P21951 | POL2 | pol epsilon | 32.788 | 32.120 | 32.599 | 34.060 | 34.697 | 33.908 | 34.221 | 32.502 | 1.719 | 0.00534 |
| P24482 | DPB2 | pol epsilon | 30.662 | 30.119 | 29.762 | 32.205 | 32.428 | 32.767 | 32.467 | 30.181 | 2.286 | 0.00176 |
| P27344 | DPB3 | pol epsilon | 29.727 | 29.220 | 29.802 | 30.097 | 30.667 | 30.219 | 30.328 | 29.583 | 0.745 | 0.04171 |
| Q04603 | DPB4 | pol epsilon | 29.726 | 29.837 | 30.183 | 31.457 | 31.945 | 31.513 | 31.638 | 29.916 | 1.723 | 0.00113 |
| P10363 | PRI1 | pol alpha / primase | 28.871 | 28.506 | 28.624 | 32.224 | 32.179 | 31.996 | 32.133 | 28.667 | 3.466 | 0.00001 |
| P13382 | POL1 | pol alpha / primase | 28.540 | 27.143 | 28.434 | 33.835 | 33.941 | 33.609 | 33.795 | 28.039 | 5.756 | 0.00023 |
| P20457 | PRI2 | pol alpha / primase | 24.065 | 23.399 | 23.569 | 31.288 | 31.523 | 31.316 | 31.376 | 23.678 | 7.698 | 0.00000 |
| P38121 | POL12 | pol alpha / primase | 28.442 | 26.615 | 28.222 | 32.713 | 32.417 | 32.267 | 32.466 | 27.760 | 4.706 | 0.00134 |
| P04786 | TOP1 | repl.-associated factors | 31.623 | 31.409 | 31.859 | 34.777 | 35.013 | 34.529 | 34.773 | 31.630 | 3.143 | 0.00008 |
| P25588 | MRC1 | repl.-associated factors | 34.933 | 35.119 | 34.831 | 37.110 | 37.461 | 37.594 | 37.388 | 34.961 | 2.427 | 0.00013 |
| P32354 | MCM10 | repl.-associated factors | 25.877 | 24.068 | 26.211 | 29.164 | 29.428 | 28.991 | 29.194 | 25.385 | 3.809 | 0.00493 |
| P32558 | SPT16 | repl.-associated factors | 34.877 | 34.614 | 34.738 | 36.865 | 37.072 | 36.909 | 36.949 | 34.743 | 2.206 | 0.00002 |
| P38766 | RRM3 | repl.-associated factors | 29.864 | 29.483 | 29.512 | 31.954 | 32.803 | 31.835 | 32.197 | 29.619 | 2.578 | 0.00143 |
| P52286 | SKP1 | repl.-associated factors | 32.071 | 31.549 | 31.974 | 33.118 | 33.277 | 33.071 | 33.155 | 31.865 | 1.291 | 0.00169 |
| P53840 | TOF1 | repl.-associated factors | 34.278 | 32.848 | 34.288 | 36.187 | 37.057 | 36.724 | 36.656 | 33.805 | 2.851 | 0.00622 |
| Q04636 | POB3 | repl.-associated factors | 33.510 | 33.260 | 33.717 | 35.748 | 35.763 | 35.759 | 35.757 | 33.496 | 2.261 | 0.00007 |
| Q04659 | CSM3 | repl.-associated factors | 31.244 | 31.570 | 31.816 | 34.122 | 34.172 | 33.994 | 34.096 | 31.543 | 2.553 | 0.00013 |
| Q08273 | HRT1 | repl.-associated factors | 30.268 | 30.085 | 30.549 | 32.061 | 32.487 | 32.454 | 32.334 | 30.301 | 2.033 | 0.00045 |
| Q08496 | DIA2 | repl.-associated factors | 33.399 | 32.530 | 32.779 | 35.750 | 35.965 | 35.351 | 35.689 | 32.903 | 2.786 | 0.00090 |
| Q12018 | CDC53 | repl.-associated factors | 32.641 | 32.967 | 33.335 | 35.063 | 35.322 | 35.407 | 35.264 | 32.981 | 2.283 | 0.00054 |
