# Supplementary material for: Unscheduled DNA replication in G1 causes genome instability and damage signatures indicative of replication collisions
Source: Nat Commun. 2022 Nov 18;13:7014. doi: 10.1038/s41467-022-34379-2 (PMC9674678; doi:10.1038/s41467-022-34379-2)
Supplement: Supplementary file 3 — Reporting Summary [file 41467_2022_34379_MOESM3_ESM.pdf]

nature portfolio

Corresponding author(s): Boris Pfander
Last updated by author(s): 10/10/2022

Reporting Summary

Nature Portfolio wishes to improve the reproducibility of the work that we publish. This form provides structure for consistency and transparency in reporting. For further information on Nature Portfolio policies, see our Editorial Policies and the Editorial Policy Checklist.

Statistics

For all statistical analyses, confirm that the following items are present in the figure legend, table legend, main text, or Methods section.

n/a	Confirmed	
☐	☒	The exact sample size (*n*) for each experimental group/condition, given as a discrete number and unit of measurement
☐	☒	A statement on whether measurements were taken from distinct samples or whether the same sample was measured repeatedly
☐	☒	The statistical test(s) used AND whether they are one- or two-sided<br>*Only common tests should be described solely by name; describe more complex techniques in the Methods section.*
☒	☐	A description of all covariates tested
☐	☒	A description of any assumptions or corrections, such as tests of normality and adjustment for multiple comparisons
☐	☒	A full description of the statistical parameters including central tendency (e.g. means) or other basic estimates (e.g. regression coefficient) AND variation (e.g. standard deviation) or associated estimates of uncertainty (e.g. confidence intervals)
☐	☒	For null hypothesis testing, the test statistic (e.g. *F*, *t*, *r*) with confidence intervals, effect sizes, degrees of freedom and *P* value noted<br>*Give P values as exact values whenever suitable.*
☒	☐	For Bayesian analysis, information on the choice of priors and Markov chain Monte Carlo settings
☒	☐	For hierarchical and complex designs, identification of the appropriate level for tests and full reporting of outcomes
☒	☐	Estimates of effect sizes (e.g. Cohen's *d*, Pearson's *r*), indicating how they were calculated

Our web collection on statistics for biologists contains articles on many of the points above.

Software and code

Policy information about availability of computer code

Data collection	Fujifilm Image Reader LAS-3000 (v2.2)<br>GE Typhoon FLA 9000 control software (v1.1)<br>MACSQuantify (v2.11)
Data analysis	bwa (v0.7.17)<br>samtools (v1.12)<br>deepTools (v3.5.1)<br>pyGenomeTracks (v3.6)<br>MACS2 (v2.2.7.1)<br>FREEC (v11.6)<br>STAR (v2.7.10a)<br>htseq-count (v2.0.1)<br>DESeq2 (v1.36.0)<br>MaxQuant (v1.5.3.54)<br>R (v4.0.3)<br>FlowJo (v10.6.2)<br>FALCOR (https://lianglab.brocku.ca/FALCOR/) (web tool, not versioned, used on Sept 7, 2020)

For manuscripts utilizing custom algorithms or software that are central to the research but not yet described in published literature, software must be made available to editors and reviewers. We strongly encourage code deposition in a community repository (e.g. GitHub). See the Nature Portfolio guidelines for submitting code & software for further information.

Data

Policy information about availability of data

All manuscripts must include a data availability statement. This statement should provide the following information, where applicable:

- Accession codes, unique identifiers, or web links for publicly available datasets
- A description of any restrictions on data availability
- For clinical datasets or third party data, please ensure that the statement adheres to our policy

All sequencing data have been deposited as raw fastq-files as well as depth-normalized bigWig-files and are available via accession number GSE208590. The mass spectrometry proteomics data have been deposited to the ProteomeXchange Consortium via the PRIDE partner repository with the dataset identifiers PXD028308 and PDX035629.

Field-specific reporting

Please select the one below that is the best fit for your research. If you are not sure, read the appropriate sections before making your selection.

☒ Life sciences ☐ Behavioural & social sciences ☐ Ecological, evolutionary & environmental sciences

For a reference copy of the document with all sections, see nature.com/documents/nr-reporting-summary-flat.pdf

Life sciences study design

All studies must disclose on these points even when the disclosure is negative.

Sample size	All data are from ensemble experiments from at least 20.000.000 budding yeast cells; for flow cytometry experiments at least 10.000 of those cells were analyzed. No sample size calculation was performed, published data of isogenic yeast strains and replication experiments show that such cell numbers are entirely large enough to reproducibly reveal attributes of yeast cell ensembles.
Data exclusions	Pre-established criteria were used to select NGS and flow cytometry data for further analysis.<br>NGS: Reads mapping to repetitive regions and ambiguously mapping reads were excluded from analysis.<br>Flow cytometry: Non-permeabilized cells, which were not stained by SYTOX green, were excluded from analysis of EdU-Cy5 fluorescence.
Replication	All experiments were performed at least twice and all attempts at replication were successful.
Randomization	Randomization was not relevant to this study since samples did not need to be allocated to specific groups.<br>In addition, an isogenic wild-type yeast strain was included in all experiments as a negative control.
Blinding	Blinding was not relevant because there was no group allocation during data collection or analysis.<br>Wherever possible, scripted analyses were used to avoid bias when quantifying data.

Reporting for specific materials, systems and methods

We require information from authors about some types of materials, experimental systems and methods used in many studies. Here, indicate whether each material, system or method listed is relevant to your study. If you are not sure if a list item applies to your research, read the appropriate section before selecting a response.

Materials & experimental systems

n/a	Involved in the study	
☐	☒	Antibodies
☐	☒	Eukaryotic cell lines
☒	☐	Palaeontology and archaeology
☒	☐	Animals and other organisms
☒	☐	Human research participants
☒	☐	Clinical data
☒	☐	Dual use research of concern

Methods

n/a	Involved in the study	
☐	☒	ChIP-seq
☐	☒	Flow cytometry
☒	☐	MRI-based neuroimaging

Antibodies

Antibodies used	anti-gammaH2A (abcam, ab181447, EPR17588), anti-Rad53 (abcam, ab104232, polyclonal), anti-RFA (Agrisera, AS07 214, polyclonal), anti-Dpb11 (Diffley lab/Pfander lab, polyclonal), anti-Sld2 (Zegerman lab, polyclonal), secondary anti-rabbit-HRP (Jackson Immuno Research, 111-035-045, polyclonal)

Validation	anti-gammaH2A, anti-Rad53, anti-RFA were validated by the manufacturers to be used for western blots with budding yeast whole cell extracts. These three antibodies as well as the antibodies against Dpb11 and Sld2 have also been used and verified in previous studies in our lab (Pfander and Diffley (2011) EMBO Journal, Reusswig et al. (2016) Cell Reports, Bantele et al. (2019) Nature Communications, Peritore et al. (2021) Molecular Cell)

Eukaryotic cell lines

Policy information about cell lines

Cell line source(s)	Yeast strains from the Pfander laboratory strain collection. All strains were constructed in the W303 background and are listed in Supplementary Table 1.
Authentication	No mammalian cell lines were used. Gene deletions and single plasmid integrations in yeast strains were confirmed by PCR, introduction of point mutations in yeast strains was confirmed by sequencing.
Mycoplasma contamination	No mammalian cell lines were used and mycoplasma do not grow in yeast cell cultures.
Commonly misidentified lines (See ICLAC register)	No commonly misidentified cell lines were used.

ChIP-seq

Data deposition

☒ Confirm that both raw and final processed data have been deposited in a public database such as GEO.

☒ Confirm that you have deposited or provided access to graph files (e.g. BED files) for the called peaks.

Data access links *May remain private before publication.*	https://www.ncbi.nlm.nih.gov/geo/query/acc.cgi?acc=GSE208590
Files in database submission	GSE208590_RAW.tar contains all depth-normalized bigWig-files (strand-separated for RPA-ChIP) as well as the BED files with called peak summits.<br>All raw FASTQ files (paired-end, 2 files per sample) are available via SRA.
Genome browser session (e.g. UCSC)	See link above; all data are publicly available via GEO.

Methodology

Replicates	n=2 for all experiments, except for "wild-type released from G1 to HU" (n=1).
Sequencing depth	±10 million reads per sample, paired-end (75 bp or 37 bp (for RPA-ChIP timecourse experiment))
Antibodies	anti-RFA (Agrisera, AS07 214)
Peak calling parameters	Reads were mapped to the budding yeast reference genome (sacCer3) using bwa (v0.7.17) and the alignments were sorted and indexed using samtools (v1.12). For RPA-ChIP-samples, reads were separated by strands at this point using samtools with the options "-f 99" and "-f 147" for the forward strand or "-f 83" and "-f 163" for the reverse strand. Afterwards, bamCoverage from deepTools (v3.5.1) was used with the options "--binSize 50 --minMappingQuality 60 --normalizeUsing CPM" to generate depth-normalized bigWig-files.<br><br>Peak calling was performed only for the analysis of origin firing in Fig S5a,b. MACS2 callpeak (v2.2.7.1) was used with default parameters in paired-end mode with a q-value of 0.1. The position of the summits of the peaks are provided as a BED file in the GEO submission.
Data quality	Quality of raw data was assessed using fastQC (v0.11.9).
Software	bwa (v0.7.17), samtools (v1.12), deepTools (v3.5.1), MACS2 (v2.2.7.1), R (v4.0.3)

Flow Cytometry

Plots

Confirm that:

☒ The axis labels state the marker and fluorochrome used (e.g. CD4-FITC).

☒ The axis scales are clearly visible. Include numbers along axes only for bottom left plot of group (a 'group' is an analysis of identical markers).

☒ All plots are contour plots with outliers or pseudocolor plots.

☒ A numerical value for number of cells or percentage (with statistics) is provided.

Methodology

Sample preparation	Briefly, yeast cells were fixed and permeabilized in 70% ethanol + 50 mM Tris-HCl pH 8.0, digested with RNase A and proteinase K and stained with 0.5 µM SYTOX green (Invitrogen, S7020).<br>To measure EdU incorporated into the DNA, cells were subjected to a click chemistry reaction using disulfo-Cy5-picolylazide (Jena Bioscience, CLK-1177) as the azide component for the reaction. Samples were subsequently washed with PBS + 10% ethanol and stained with 0.5 µM SYTOX green.<br><br>See Methods section for more detail.
Instrument	Miltenyi Biotec MACSquant analyzer 10
Software	FlowJo (v10.6.2)
Cell population abundance	No gating was performed.
Gating strategy	No gating was performed.

☐ Tick this box to confirm that a figure exemplifying the gating strategy is provided in the Supplementary Information.
